# Imposed expiratory resistance, dynamic hyperinflation and locomotor power and fatigue

**DOI:** 10.1113/EP092818

**Published:** 2025-07-30

**Authors:** Jonathan Cunha, Antoinette Domingo, Fred W. Kolkhorst, Harry B. Rossiter, Daniel T. Cannon

**Affiliations:** ^1^ School of Exercise and Nutritional Sciences San Diego State University San Diego California USA; ^2^ Department of Surgery University of Michigan Ann Arbor Michigan USA; ^3^ School of Physical Therapy San Diego State University San Diego California USA; ^4^ Institute of Respiratory Medicine and Exercise Physiology, Division of Respiratory Critical Care Physiology & Medicine The Lundquist Institute at Harbor‐UCLA Medical Center Torrance California USA

**Keywords:** exercise intolerance, isokinetic

## Abstract

Expiratory flow limitation results in dynamic hyperinflation, dyspnoea and premature exercise intolerance. We aimed to measure whether expiratory resistance reduces locomotor power via limiting maximal voluntary motor activity, exacerbating muscle fatigue, or both. Healthy volunteers (*n* = 14; 23 (3) years) performed a series of very heavy‐domain constant power cycling exercise tests with and without an imposed expiratory flow resistance (7 cmH_2_O/L/s). The decline in maximal evocable isokinetic power at intolerance during each experimental condition was apportioned to: (1) the power equivalent from a reduction in maximum voluntary muscle activation (termed ‘activation fatigue’); and (2) the deficit in expected power at a given isokinetic muscle activity (muscle fatigue). Imposed expiratory resistance reduced exercise tolerance (487 (145) vs. 575 (137) s; 95% confidence interval of the difference (CI_diff_) 52, 125 s; *P =* 0.0002). At isotime‐control, imposed expiratory resistance resulted in a greater decline in inspiratory reserve volume (CI_diff_ 0.20, 0.94 L; *P =* 0.007), and increased dyspnoea (Borg CR‐10; CI_diff_ 0.7, 3.0; *P =* 0.006) than without. Muscle fatigue was unaffected (CI_diff_ −20, 17 W; *P =* 0.873), but activation fatigue was greater with expiratory resistance (CI_diff_ 1, 49 W; *P =* 0.044) and related to the reduction in inspiratory reserve volume (*r* ^2 ^= 0.53; *P =* 0.028). As a result, locomotor power reserve was reduced with expiratory resistance (253 (83) vs. 201 (92) W; CI_diff_ −10, 113; *P =* 0.09). Imposed expiratory resistive loading initiated a cascade of abnormal lung mechanics and symptoms. These abnormalities conflate to reduce exercise tolerance through limiting maximal voluntary motor activity.

## Abstract (highlights)

- **What is the central question of this study?**
  Resistance to expiration, such as in asthma and chronic obstructive lung disease, causes abnormal lung function and intolerance to exercise: does abnormal lung mechanics directly affect locomotor fatigue?
- **What is the main finding and its importance?**
  Imposed expiratory resistance initiates a cascade of abnormal lung mechanics and symptoms, which conflate to reduce exercise tolerance through limiting maximal voluntary motor activity.

## 1 INTRODUCTION

Fatigue is a reduction in muscle force or power output that is reversible after rest (Bigland‐Ritchie & Woods, 1984). Mechanisms of fatigue may arise at any step from the motor cortex to the actin–myosin interaction. Most broadly, fatigue can be separated into peripheral and central components. Peripheral fatigue generally encompasses biochemical changes in the muscle metabolic environment that result in reduced responses to excitation, while central fatigue is the failure of the central nervous system to drive the motor neurons (Amann, 2011). The intramuscular mechanisms of fatigue result primarily from the depletion of energy stores (phosphocreatine, glycogen) and accumulation of metabolites (inorganic phosphate, lactate, H^+^, ADP, H_2_O_2_, O_2_^−^) that interfere with energy provision and cross‐bridge cycling (Kent‐Braun et al., 2012). These intramuscular disturbances lead to attenuation of force or power at a given neural excitation (Grassi et al., 2015). Conversely, central mechanisms of fatigue include sensory feedback from afferents and chemoreceptors, for example, from locomotor or respiratory muscle, lung or airway, which modulate motor cortex output and reduce central motor drive (Meeusen & Roelands, 2010, 2018; O'Donnell et al., 2007).

When fatigue accumulation is progressive during exercise, intolerance will result when the maximal power output falls below the requirement to sustain the task. In ageing or chronic disease, where fatigue resistance and exercise tolerance are poor, this may lead to limitation of the activities of daily living. Given that exercise tolerance is the strongest predictor of mortality (Kokkinos et al., 2022; Myers et al., 2002), a better understanding of the mechanisms of, and strategies to mitigate, fatigue and exercise intolerance are pressing.

Inspiratory and expiratory muscle fatigue can exacerbate limb locomotor fatigue, exercise tolerance and perceptions of leg discomfort (Taylor & Romer, 2008; Wuthrich et al., 2013). Fatigue of the respiratory muscles increases sympathetic vasoconstrictor outflow, and through this respiratory‐muscle metaboreflex the blood flow to locomotor skeletal muscle is compromised. The cascading effect may result in locomotor muscle fatigue, worsened locomotor fatigue perceptions, reduced motor output and poor exercise tolerance (Romer & Polkey, 2008). Conversely, training of the inspiratory muscles can improve exercise tolerance, also likely through reduced dyspnoea, improved blood flow to the limbs and faster dynamics in oxidative metabolism (Bailey et al., 2010; Ramsook et al., 2017). Many of these study designs incorporate fatiguing bouts of inspiratory or expiratory muscle contractions, voluntary exercise and the interpolated twitch technique to understand the interplay between respiratory and locomotor muscles. However, none incorporate imposed expiratory resistive loading during exercise to induce dynamic hyperinflation, as seen in chronic obstructive pulmonary disease (COPD). Dynamic hyperinflation is a result of expiratory flow limitation that requires a leftward shift on the flow–volume relationship to generate the flow demanded by the ventilatory needs. In other words, the operating lung volumes increase to meet the gas flow demands of the task. Also, no other studies examine locomotor fatigue using maximal voluntary power measurements to investigate whether maximal voluntary power decays enough to limit the exercise task. Using a combination of constant power and maximal voluntary isokinetic power assessment, we aimed to measure how resistive loading may result in abnormal lung mechanics, skeletal muscle fatigue and exercise intolerance – such as in conditions characterized by expiratory flow limitation (Vogiatzis & Zakynthinos, 2013).

To better understand how expiratory flow limitation initiates the cascade culminating in exercise intolerance, we used the musculo‐cardio‐pulmonary exercise testing (mCPET) technique to measure (1) total reduction in locomotor power, (2) skeletal muscle fatigue (reduced power at a given motor activity), and (3) ‘activation’ fatigue (power equivalent from reduced evocable motor activity) during dynamic whole‐body exercise in healthy humans with and without imposed expiratory resistive loading.

We hypothesized that (1) imposed expiratory resistance reduces locomotor power and exercise tolerance, (2) expiratory resistance exacerbates activation fatigue, and (3) activation fatigue is most closely related to dynamic hyperinflation and dyspnoea.

## 2 METHODS

### 2.1 Participants

Participants were recruited by word‐of‐mouth and through printed advertisements. Inclusion criteria were men and women, 18–45 years old, who engaged in regular exercise (three times per week for the previous 6 months). Exclusion criteria were current or history of smoking and cardiopulmonary disease. The San Diego State University Institutional Review Board approved the study protocol and all supplements (SDSU IRB no. 1928098). The study conformed to the standard set by the *Declaration of Helsinki*, except for registration in a database. Participants provided written informed consent and were screened for cardiovascular risks with the Physical Activity Readiness Questionnaire (PAR‐Q) prior to taking part in the study.

### 2.2 Exercise protocols

**FIGURE 1:**
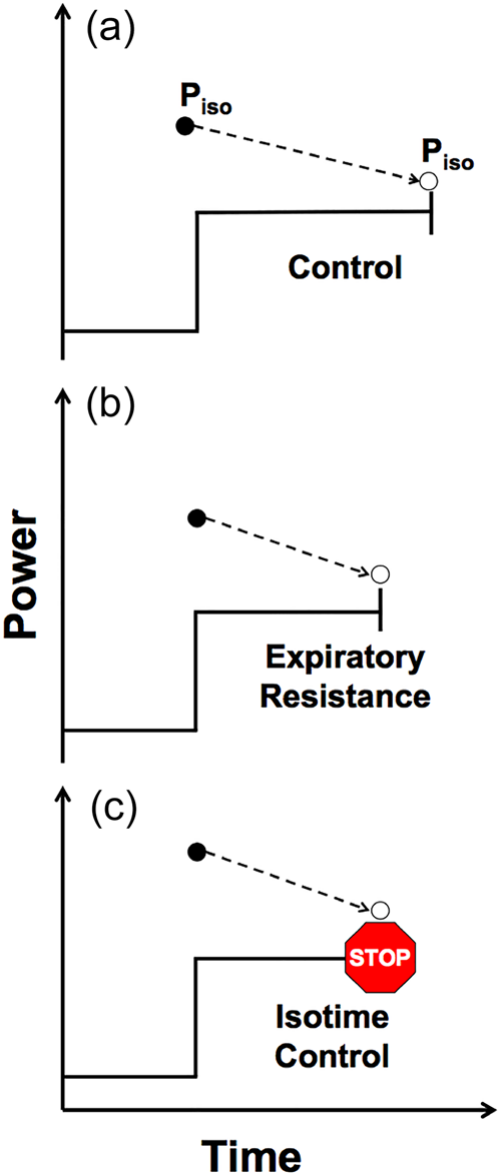
Schematic representation of the three experimental conditions. (a, b) Maximal isokinetic power (*P*_iso_) was measured prior to and immediately following the limit of tolerance to constant power exercise without (a) and with (b) expiratory resistance. (c) Isotime‐control exercise was terminated and *P*_iso_ measured at the equivalent tolerance time to expiratory resistance.

Participants completed four laboratory visits, each separated by 48 h, consisting of incremental (visit 1) or constant power exercise tests (visits 2–4). A schematic representation of the constant power exercise tests is presented in Figure 1. Volunteers were asked to abstain from alcohol and strenuous exercise in the preceding 24 h before each visit.

**FIGURE 2:**
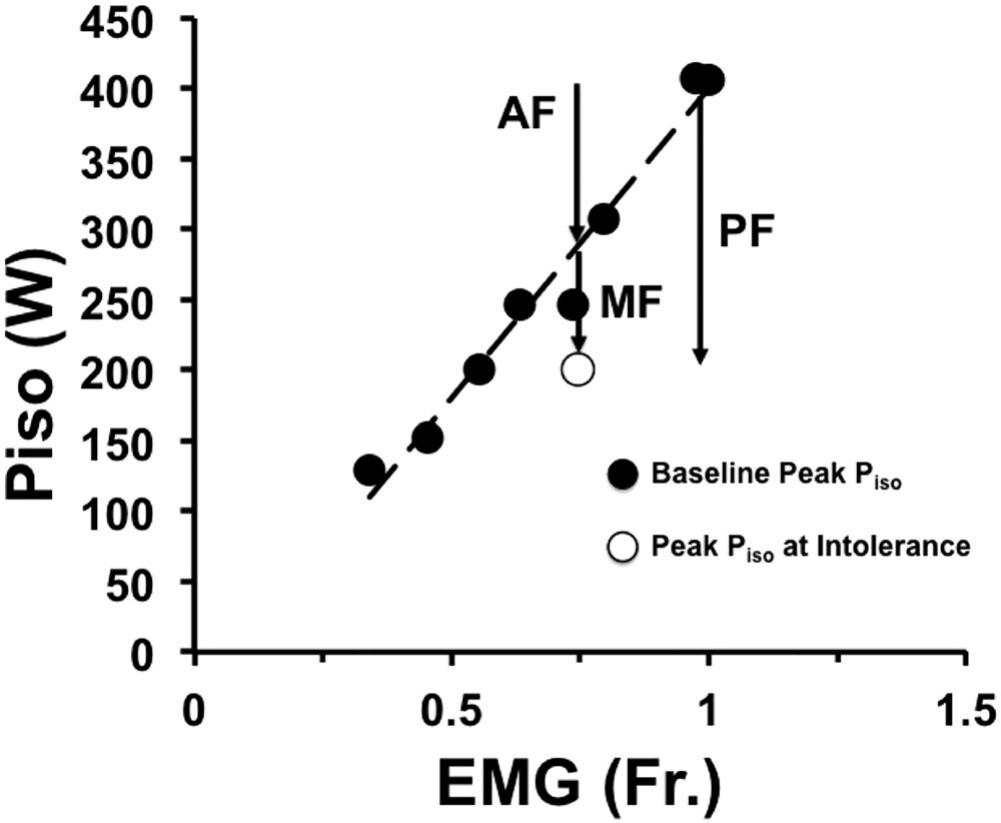
Relationship between fractional (Fr.) electromyographic activity (EMG) and isokinetic power (*P*_iso_). The data include the baseline (fatigue‐free) measurements (●) and the maximal‐effort measurement at the limit of tolerance (○). All measurements were taken at 70 rpm. Performance fatigue (PF) arrow represents the total reduction in power generation immediately at the limit of ramp‐incremental exercise. Activation fatigue (AF) represents the proportion of performance fatigue resulting from a reduced maximal evocable muscle activity. Muscle fatigue (MF) arrow represents the proportion of performance fatigue that can be attributed to MF, that is, a lower power than expected for the measured EMG.

Prior to all exercise tests, participants completed a series of short (∼5 s), variable‐effort isokinetic bouts to characterize the baseline EMG and isokinetic power relationship (EMG–*P*_iso_) at 70 rpm (Figure 2). Participants were instructed to give variable efforts of approximately 25%, 50%, 75% and 100% of maximum cycling power. Each bout lasted approximately 5 s and was followed by at least 30 s of 0 W cycling. This process was repeated for a total of eight isokinetic bouts (Figure 2) (Cannon et al., 2016; Coelho et al., 2015).

Following a rest period of 5–10 min, participants completed either the ramp incremental or constant power test. During the ramp and constant power tests, the ergometer was set to the cadence‐independent (hyperbolic) mode. The ramp incremental test consisted of approximately 3 min at rest, 2 min of 25 W cycling, and a ramp phase of 20–25 W/min until reaching the limit of tolerance. The limit of tolerance was defined as failure to maintain a minimum pedalling cadence of 60 rpm for at least 5 s, despite strong verbal encouragement. At the limit of tolerance, the ergometer was switched from hyperbolic mode to isokinetic mode and participants were strongly encouraged to provide a final maximal effort for ∼5 s. Primarily, this isokinetic effort was used as a familiarization trial.

The constant power tests consisted of approximately 3 min of rest, 2 min of 25 W cycling, and a constant power phase until reaching the limit of tolerance. The constant power was selected at 70% of the difference between estimated lactate threshold (Beaver et al., 1986) and ${\dot{V}}_{O_{2}\mathrm{peak}}$. These trials were done in the control condition and with an imposed expiratory resistance of 7 cmH_2_O/L/s in a counterbalanced order (Erram et al., 2021). Expiratory loading was achieved by using an inspiratory muscle training device (Threshold IMT, Respironics, Pittsburgh, PA, USA) installed in reverse in the flow‐sensor. At the limit of tolerance, the ergometer was switched to isokinetic mode and subjects were encouraged to provide a final maximal effort for ∼5 s, similar to the familiarization during the ramp incremental test in visit 1 (Figure 1). During the final laboratory visit, the participants completed what we termed the isotime‐control condition. That is, the exercise test was terminated after completing the duration equal to that achieved in the expiratory resistance trial, but under the control condition. At the completion of this predetermined duration, the ergometer was switched to isokinetic mode and participants were given strong verbal encouragement to provide a final maximal effort for ∼5 s (Figure 1c).

### 2.3 Ergometry

The computer‐controlled electromagnetically braked cycle ergometer (Excalibur Sport PFM, Lode BV, Groningen, Netherlands) was instrumented with force transducers in the bottom bracket spindle. Left and right torque (N m) was measured independently (peak force 2000 N, <0.5 N resolution and measurement uncertainty of <3%). Instantaneous angular velocity of the crank (rad/s) was measured with a resolution of 2° using three independent sensors sampling in series (measurement uncertainty of <1%). During isokinetic efforts, power was calculated every 2° from torque and angular velocity measurements. There was no systematic difference in the power output between the left and right cranks. Therefore, *P*_iso_ was calculated from power on left crank averaged over three crank revolutions and was paired with an EMG datum from the same leg (described below). Where appropriate, crank power is reported as two times one‐leg to allow for direct comparison with power output measured at the flywheel (which, in effect, is a sum of both legs, both positive and negative power, throughout the pedal stroke).

### 2.4 Electromyography

Surface EMG activity was measured in four muscles of the left leg: vastus lateralis, rectus femoris, vastus medialis and lateral gastrocnemius. Placement sites were shaved, abraded with gauze and cleaned with warm, soapy water. Wireless‐transmitting Ag bipolar parallel‐bar surface electrodes were placed over the muscle belly (Trigno Wireless System, Delsys Inc, Boston, MA). Electrodes were placed over the vastus lateralis halfway between the greater trochanter and the patella, over the rectus femoris halfway between the anterior superior iliac spine and the patella, over the vastus medialis two‐thirds of the distance from the anterior superior iliac spine to the medial epicondyle, and over the lateral gastrocnemius one‐third of the distance between the popliteal fossa and the lateral malleolus. The longitudinal axis of the electrode was aligned parallel to the long axis of the muscle.

EMG signals were differentially amplified and sampled at 2000 Hz with 16‐bit resolution. Each sensor had a signal bandwidth of 20–450 Hz and common mode rejection ratio of 80 dB (EMGWorks Acquisition, Delsys Inc, Boston, MA, USA). Raw EMG data were analysed using custom‐written routines in MATLAB (MathWorks, Natick, MA, USA). During post‐processing, signals were filtered with a fourth‐order Butterworth bandpass filter (10–500 Hz) and smoothed via root mean square (RMS) with a 200‐sample moving window. The peak activity (*V*; from the 200‐sample RMS) from each crank revolution was used as an estimate of muscle activity. Three consecutive isokinetic crank revolutions that were appropriately constrained at the desired angular velocity were identified in the output from the cycle ergometer. The mean *P*_iso_ from these three revolutions was paired with the time‐aligned RMS EMG. The RMS EMG value was generated from the mean of the four muscles of the left leg. The muscle selection reflected the approximate‐weighted power contributions during cycling from knee extension/flexion and plantar flexion (Ericson et al., 1986).

Characterization of the baseline EMG–*P*_iso_ relationship is specific to the electrode placement (skin preparation, conduction, etc.), and therefore was done for each laboratory visit. This measure provides a reference for the expected power production from a given EMG activity. The data were fit using linear regression after EMG activity was normalized to the maximum activity during the baseline phase.

### 2.5 Fatigue characterization

For characterization of the EMG–*P*_iso_ relationship the RMS EMG values were normalized to the visit maximum. The baseline linear relationship between power production and EMG activity (measurement of baseline EMG–*P*_iso_) was characterized using least‐squares regression. Measurements made at the limit of tolerance for ramp incremental exercise were used to calculate three fatigue measurements (each expressed in W). Performance fatigue was the reduction in *P*_iso_ (W) from the baseline (fatigue‐free) maximum. The proportion of performance fatigue resulting from activation fatigue was calculated from the power equivalent of the reduction in RMS EMG activity, using the baseline linear regression between EMG and *P*_iso_ at 70 rpm. Muscle fatigue was calculated from the balance (MF = PF − AF; with lower bounds constrained at 0 W), that is, the deviation in power from the baseline EMG–*P*_iso_ relationship at the measured EMG value (for a graphical representation for these indices, see Figure 2) (Cannon et al., 2016; Coelho et al., 2015).

### 2.6 Cardiopulmonary measurements

Respired gases and ventilation were measured breath‐by‐breath with a commercial metabolic measurement system (VMax Encore, CareFusion, San Diego, CA, USA). The system was calibrated immediately prior to each testing session. A 3 L syringe (Hans Rudolph Inc., Shawnee, KS, USA) was used to calibrate the mass flow sensor from ∼0.2 to 8.0 L/s, mimicking flow rates expected at rest and during exercise. The CO_2_ and O_2_ analysers were calibrated using gases of known concentrations (O_2_ 26.0% and 16.0%; CO_2_ 0.0% and 4.0%). Inspiratory capacity (IC) was measured at baseline, isotime and the limit of tolerance in duplicate. The IC measurement consisted of spirometry interleaved during exercise. When serial end‐expiratory lung volume (EELV) was stable, the volunteer was queued to take a maximal inspiration to total lung capcity (TLC). The IC volume was measured as the difference between EELV and TLC, while the inspiratory reserve volume (IRV) was IC – tidal volume (*V*_T_). *V*_T_ matched for each IC manoeuvre was chosen as a 20 s mean preceding the spirometry manoeuvre. At baseline, isotime and the limit of tolerance, dyspnoea and leg effort were assessed using the modified Borg scale (CR‐10) followed by an IC manoeuvre.

### 2.7 Near‐infrared spectroscopy

Near‐infrared spectroscopy (NIRS) was used to measure tissue oxygenation in the frontal lobe and the vastus lateralis during exercise. Changes in oxygenation can be determined from the absorption characteristics of NIR light by chromophores in small capillaries, arterioles and venules. As the light absorption is so high in larger blood vessels, their contribution to the NIRS signal is minimal, whereas photon migration through the smaller vasculature allows for chromophore concentration changes to be detected (Chance et al., 1992; Liu et al., 1995). The measurement technique relies on the known absorption characteristics of oxygenated haemoglobin + myoglobin (O_2_Hb) and deoxygenated haemoglobin + myoglobin (HHb) when NIR light is directed into tissue. Additionally, as the absorption coefficients are common only at ∼810 nm (but not at other wavelengths measured) for O_2_Hb and HHb, the total Hb under interrogation can be estimated, providing an indication of haem concentration in the muscle tissue.

The NIRS device (NIRO‐200, Hamamatsu Photonics KK, Hamamatsu, Japan) consisted of a laser diode light source, and a photodiode to detect the returned NIR light after passing through the tissue under interrogation. The NIRS probe (consisting of one fibre optic emission optode and two detection optodes) was secured over the vastus lateralis muscle (12 cm above the patella) using double‐sided adhesive tape and an elastic bandage to minimize ambient light contamination and movement of the probes. The probes were enclosed in a black rubber housing with a fixed emission/detection distance (detectors were 4 and 5 cm from the emitter) for measurement of the relative absorbance of each chromophore by spatially resolved spectroscopy (SRS). Source light was provided at three wavelengths (775, 810 and 850 nm) and detection sampled at 2 Hz to calculate the tissue oxygenation index (TOI). The SRS method is thought to allow signal loss, due to light scatter, to be better accounted for in calculation of TOI ([HbO_2_]/[HbO_2_ + HHb], expressed as a percentage) and the signal is therefore proportional to chromophore concentration.

### 2.8 Statistical analyses

Means were compared, where appropriate, with a dependent Student's *t*‐test. Primarily these variables include tolerance time, power outputs, measures of fatigue, isokinetic power, gas exchange, ventilation and lung volumes. Relationship strength was reported with Pearson's correlation coefficient, such as the relationships between measurements of fatigue and ventilation or spirometry. Data are presented as means (SD), and, where appropriate, the 95% confidence interval of the difference (CI_Diff_) is included. Sample size was determined (GPower 3.1) based on the primary outcome, muscle fatigue (in W) at ‘iso‐time’. That is, the amount of fatigue accumulated at the same time during control and added expiratory resistance. Fifteen volunteers are needed to detect an effect size (Cohen's *d*) of 0.7 (taken from pilot data of COPD patients; unpublished data), α = 0.05, power = 0.8. Outputs: noncentrality parameter = 2.7, critical *t* = 1.8, degrees of freedom = 14, *n* = 15, power = 0.82.

## 3 RESULTS

### 3.1 Ramp‐incremental exercise

Fourteen apparently healthy and physically active adults (23 (3) years, 170 (8) cm, 68.7 (12.5) kg, seven women, seven men) volunteered. During the incremental protocol, participants achieved a peak ramp power of 258 (55) W, a peak ${\dot{V}}_{O_{2}}$ of 3.74 (0.89) L/min, and lactate threshold was estimated at 2.05(0.45) L/min.

### 3.2 Baseline EMG–*P*_iso_

The baseline isokinetic power–EMG relationship was linear (*r* ^2^ = 0.96 (0.02)). Participants achieved a single leg (left crank arm to match EMG data) maximum baseline isokinetic power of 342 (108) W. Thus, the twice one‐leg baseline isokinetic power delivered at the chainring was 684 (216) W.

### 3.3 Constant power exercise tolerance

**FIGURE 3:**
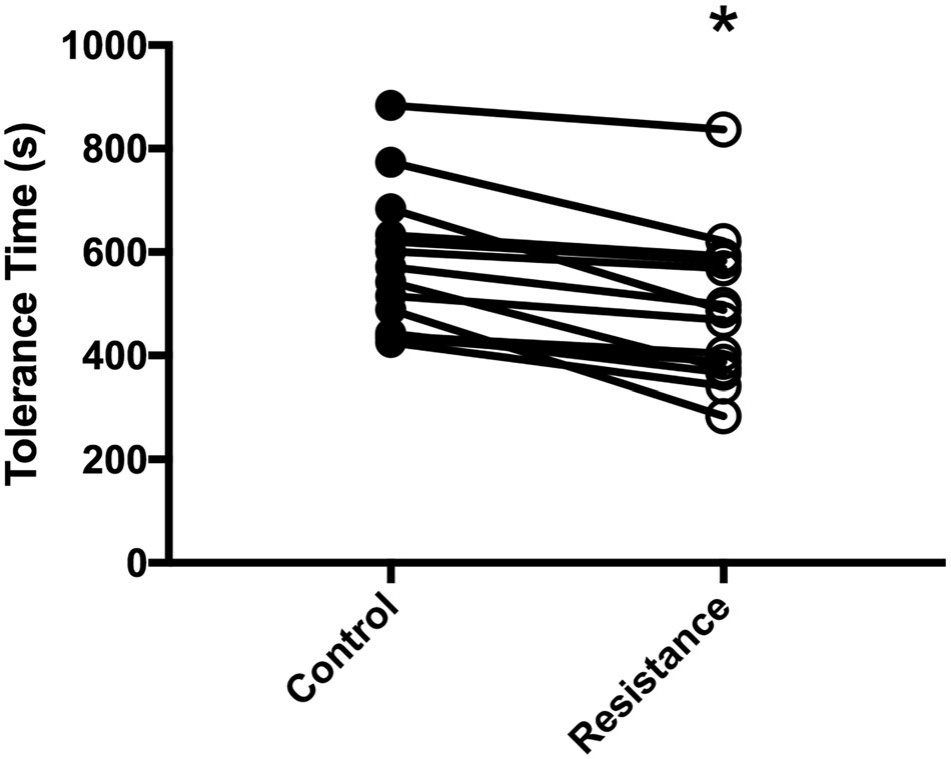
Time to intolerance with and without expiratory resistance. Expiratory resistance decreased time to limitation (487 (145) versus 575 (137) s; *t(13) = 5.21; *P *< 0.001; CI_diff_ 52, 125 s; *n* = 14).

Mean constant power was 193 (47) W. Time to intolerance was reduced ∼15% with expiratory resistance compared to the control trial (487 (145) vs. 575 (137) s; *t*(13) = 5.21, *P =* 0.0002; CI_diff_ 52, 125 s; Figure 3). There was no difference (*F*(2, 33) = 0.18; *P =* 0.84) in ${\dot{V}}_{O_{2}\mathrm{peak}}$ during constant power trials (control: 3.50 (0.78) L/min; resistance: 3.58 (0.99) L/min; isotime‐control: 3.54 (0.89) L/min).

### 3.4 Effects of expiratory resistance on measures of fatigue

**FIGURE 4:**
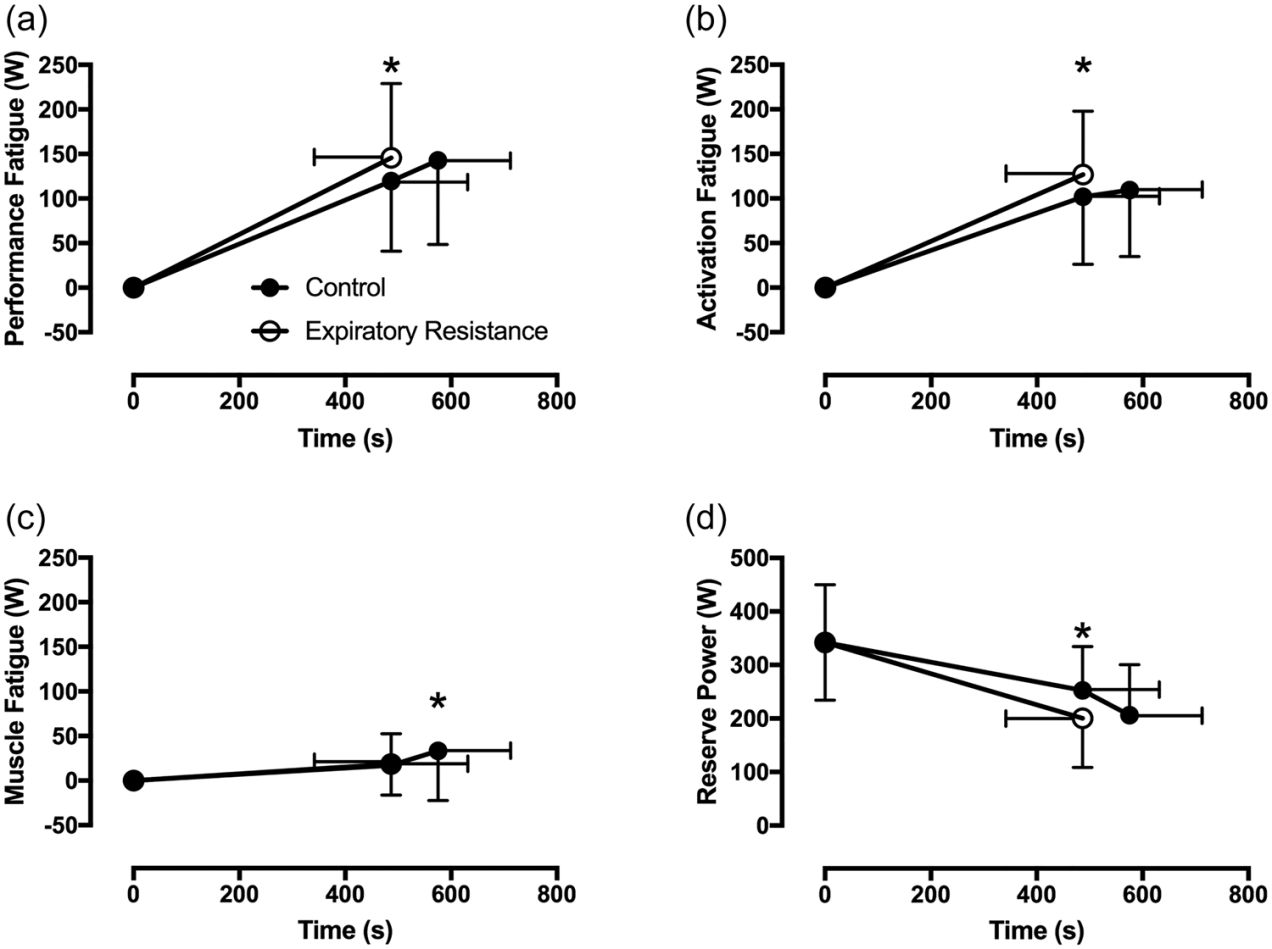
Indices of fatigue and locomotor power reserve. (a) Performance fatigue was greater at the limit of tolerance with expiratory resistance versus isotime‐control (*P = 0.09; CI_diff_ −5, 57 W; *n* = 14). (b) Activation fatigue was greater with expiratory resistance versus isotime‐control (*P < 0.05; *n* = 14). (c) Muscle fatigue was not different at the limit of tolerance with expiratory resistance at isotime‐control. However, it was greater at the limit of tolerance in the control condition versus expiratory resistance (*P < 0.05; *n* = 14). (d) Locomotor power reserve was lower with expiratory resistance versus isotime‐control (*P = 0.09; *n* = 14, CI_diff_ −10, 113).

Performance fatigue was greater at the limit of tolerance with expiratory resistance versus isotime‐control (146 (83) vs. 120 (79) W; *t*(13) = 1.82, *P =* 0.09; CI_diff_ −5, 57 W; Figure 4a). Activation fatigue was greater with expiratory resistance versus isotime‐control (127 (71) vs. 102 (76) W); *t*(13) = 2.23, *P =* 0.044; CI_diff_ 1, 49 W; Figure 4b). Muscle fatigue was not different at isotime (19 (34) vs. 17 (34) W; *t*(13) = 0.16, *P =* 0.873; CI_diff_ −17, 20 W Figure 4c). Muscle fatigue was greater, however, at the limit of tolerance in control compared to the limit of tolerance with expiratory resistance (33 (56) vs. 19 (34) W; *t*(13) = 2.13, *P =* 0.053; CI_diff_ 0, 30 W; Figure 4c).

### 3.5 Effects of expiratory resistance on locomotor power reserve

Unsurprisingly, power reserve, the difference in task power and the maximal 5 s isokinetic power at intolerance, was greater at isotime‐control versus expiratory resistance (201 (92) versus 253 (83) W; *t*(13) = −1.819, *P =* 0.09; CI_diff_ −10, 113 W; Figure 4d). However, leg effort (rating of perceived exertion (RPE)) was not different between expiratory resistance and isotime‐control (8.5 (1.6) vs. 8.0 (1.7); *t*(11) = 0.944, *P =* 0.183). Power reserve at exercise intolerance was similar in control and expiratory resistance conditions (206 (94) vs. 200 (92) W).

### 3.6 Effects of expiratory resistance on lung mechanics

**FIGURE 5:**
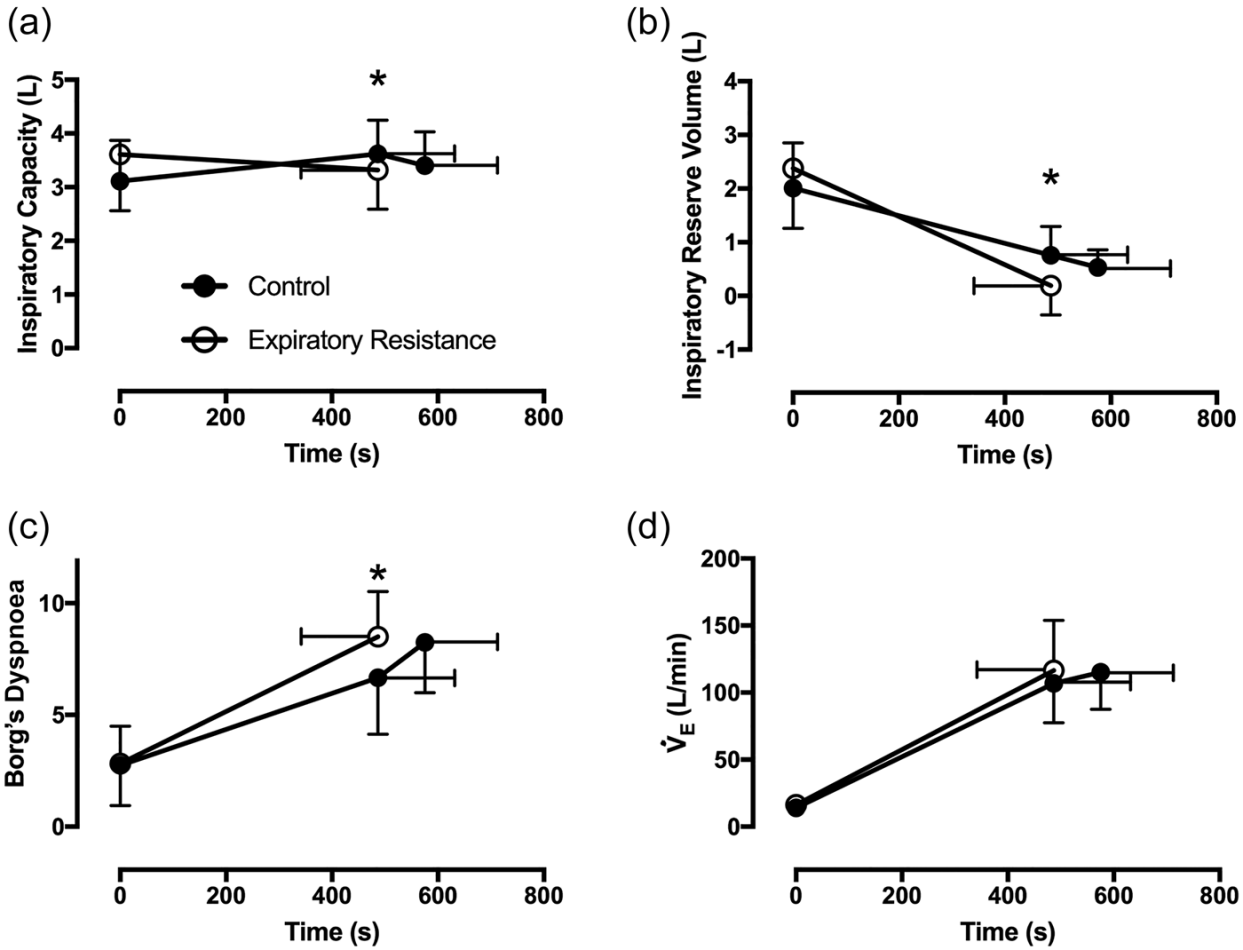
Measures of dynamic hyperinflation, dyspnoea and ventilation. (a) Inspiratory capacity reduced with expiratory resistance at isotime (*P < 0.05; *n* = 9). (b) Expiratory resistance reduced inspiratory reserve volume at isotime (*P < 0.05; *n* = 9). (c) Expiratory resistance resulted in greater dyspnoea at isotime (*P < 0.05; *n* = 12). (d) Ventilation was not different at the limit of tolerance or at isotime.

**FIGURE 6:**
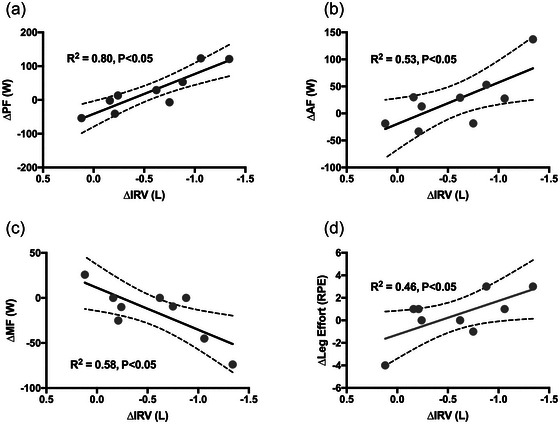
Relationships between indices of fatigue and dynamic hyperinflation at the isotime comparison. All differences (∆) at isotime are plotted as (∆ = expiratory resistance intolerable limit – isotime control). ∆IRV plotted in reverse to show worse hyperinflation to the right. Dashed lines are 95% confidence limits. (a) The ∆PF at isotime was related to the ∆IRV (*R* ^2^ = 0.80, *P *< 0.05), such that more PF was related to more hyperinflation. (b) The ∆AF at isotime was related to the ∆IRV (*R* ^2^ = 0.53, *P *< 0.05), such that AF was related to more hyperinflation. (c) ∆MF was related to ∆IRV (*R* ^2^ = 0.58, *P *< 0.05), such that less MF occurred with more hyperinflation. (d) ∆Leg effort (RPE units from 1 to 10) was related to ∆IRV (*R* ^2^ = 0.46, *P *< 0.05), such that more leg effort was related to more hyperinflation.

Expiratory resistance caused dynamic hyperinflation during exercise. IC with expiratory resistance was less versus isotime‐control (3.32 (0.73) vs. 3.62 (0.63) L; *t*(8) = −4.37, *P =* 0.007; CI_diff_ 0.14, 0.45 L; Figure 5a). Mean IRV with expiratory resistance was less versus isotime‐control (0.19 (0.56) vs. 0.76 (0.54) L; *t*(9) = −3.55, *P =* 0.007; CI_diff_ 0.20, 0.94 L; Figure 5b). Dyspnoea was greater with expiratory resistance versus isotime‐control (8.5 (2.0) vs. 6.7 (2.5); *t*(11) = 3.432, *P =* 0.006; CI_diff_ 0.7, 3.0; Figure 5c), with no difference in the minute ventilation (Figure 5d). Performance fatigue was related to the difference in IRV at isotime (*r* ^2 ^= 0.80, *P =* 0.001; Figure 6a), such that greater performance fatigue was related to greater hyperinflation. The same was true for activation fatigue (*r* ^2 ^= 0.53, *P =* 0.028; Figure 6b). Similar to IC, the difference in IRV at isotime was not correlated with dyspnoea (*r* ^2 ^= 0.18, *P =* 0.48). Contrary to our hypothesis, dyspnoea was also unrelated to activation fatigue (*r* ^2^ = 0.06, *P =* 0.5). Interestingly, the difference in muscle fatigue was negatively related to difference in IRV at isotime (*r* ^2^ = 0.58, *P =* 0.02; Figure 6c), such that less muscle fatigue occurred with greater hyperinflation. Leg effort in Borg units was related to the difference in IRV at isotime, such that greater hyperinflation was associated with greater leg effort (*r* ^2 ^= 0.46, *P =* 0.045; Figure 6d).

### 3.7 Brain and locomotor muscle oxygenation

**FIGURE 7:**
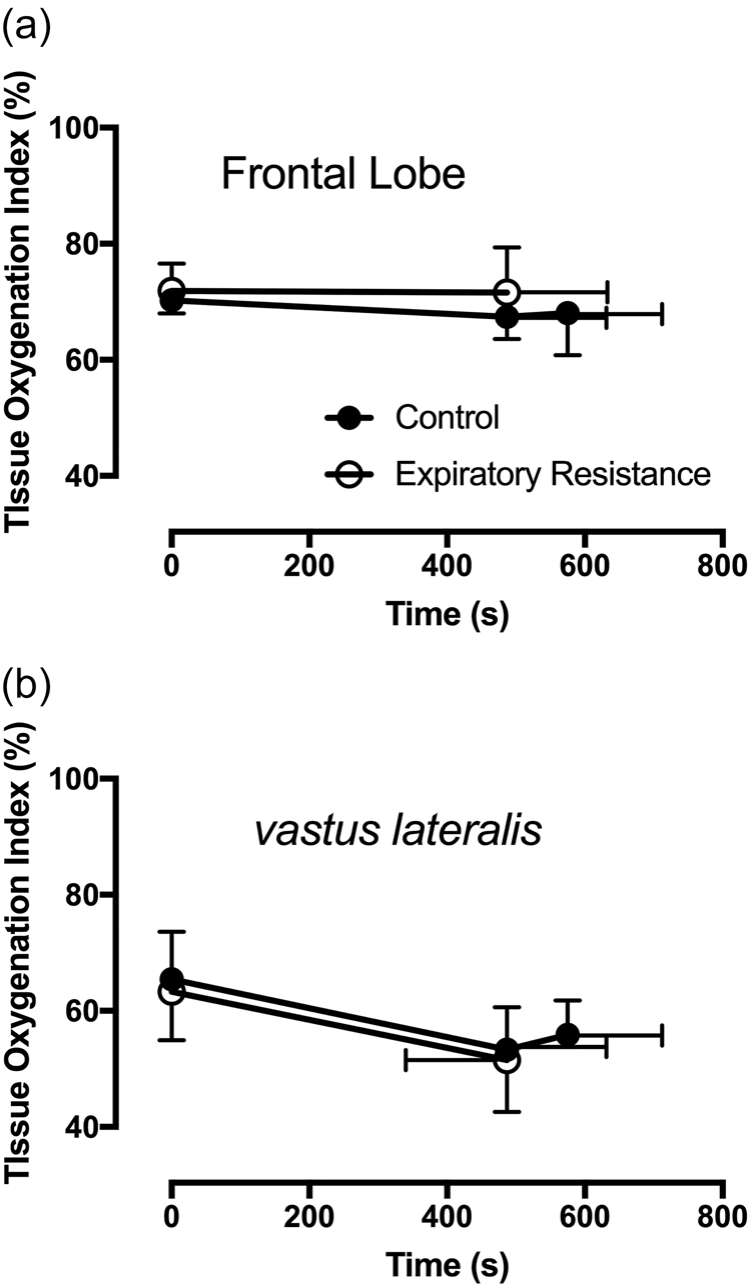
Tissue oxygenation measured via near‐infrared spectroscopy. (a) Brain oxygenation measured at the left frontal lobe. (b) Vastus lateralis oxygenation. No differences were detected at either site at isotime or intolerance.

Frontal lobe and vastus lateralis oxygenation, measured via NIRS, were not different at isotime or at the limit of tolerance with expiratory resistance (Figure 7).

## 4 DISCUSSION

We aimed to determine whether the detrimental cascade of imposed expiratory resistance, leading to reduced neuromuscular performance and exercise intolerance, could be explained by exacerbated muscle fatigue, reduced maximal voluntary cycling power, or a combination of the two. As expected, there was a reduction in time to intolerance with added expiratory resistance (∼15%; Figure 3), and a reduction in maximal voluntary cycling power at isotime (18%; Figure 4a). We saw a reduction in inspiratory capacity and inspiratory reserve volume at isotime (Figure 5a,b), indicating that we were successful in inducing dynamic hyperinflation in healthy humans using an imposed expiratory resistance of 7 cmH_2_O/L/s. We found greater activation fatigue with added expiratory resistance at isotime, without exacerbation of skeletal muscle fatigue (Figure 4b,c). Additionally, the difference in activation fatigue at isotime was closely correlated to the reduction in inspiratory capacity and inspiratory reserve volume (Figure 6b). However, there was no correlation between the difference in activation fatigue and the difference in dyspnoea at isotime. While we can only speculate about these mechanisms in patients with obstructive disease, it seems likely that hyperinflation can lead to a similar cascade that we measured in healthy people. That is, the reduced inspiratory reserve volume may lead to activation fatigue of some ‘central’ origin that limits voluntary locomotor power and contributes to exercise intolerance. This seems to happen independent of the muscle power elicited at a given motor activity. If the hyperinflation and neuromuscular fatigue cascade behaves the same way in patients with COPD, for example, there may be important clinical implications. For example, in COPD the mechanism by which supplemental O_2_ increases exercise tolerance may be more closely related to the attenuation of central fatigue mechanisms related to the reduction in ventilatory demand and hyperinflation than to amelioration of peripheral (intramuscular) fatigue initiated by, for example, the respiratory muscle blood flow steal effect. This could even be the case in individuals who are normoxic at rest and during exercise or where supplemental O_2_ is not indicated. These experiments need to be completed, however, to confirm whether activation fatigue can be improved with supplemental O_2_, or other interventions in patients with pulmonary obstruction.

### 4.1 Effect of abnormal lung mechanics on exercise tolerance

Our participants had similar dyspnoea scores at the limit of tolerance between expiratory resistance and control. However, the progression of dyspnoea with expiratory resistance was more rapid and therefore greater at isotime (Figure 5c). By comparison, reduced total lung capacity using chest wall strapping, and therefore restricted ventilation, in healthy humans reduced exercise tolerance by ∼27% (O'Donnell et al., 2000). Alternatively, by pharmacologically reducing EELV and alleviating dynamic hyperinflation in patients with COPD, there was a mean 58% increase in endurance time (O'Donnell et al., 2004). Our findings further demonstrate that abnormal exercise lung mechanics (O'Donnell et al., 2001) induced by an imposed expiratory resistance in healthy people also results in impaired exercise tolerance.

The importance of dynamic hyperinflation in contributing to the intensity of dyspnoea or exercise intolerance has been difficult to determine (Aliverti et al., 2004; Guenette et al., 2012). While we were able to induce dynamic hyperinflation with imposed expiratory resistance in healthy humans, it may not fully explain the reduced exercise tolerance. COPD patients who exhibit dynamic hyperinflation do not necessarily have increased exertional dyspnoea or greater exercise intolerance when compared with FEV_1_‐matched COPD patients who do not hyperinflate (Guenette et al., 2012). Instead, approaching a minimal IRV value (0.5 L) strongly influences dyspnoea and exercise intolerance independent of ‘acute on chronic’ dynamic hyperinflation (Guenette et al., 2012). While closely related, the two concepts of a reduced IC and IRV have different implications. IC shows the capacity for each breath, but it does not consider the operating lung volume. In other words, an operating lung volume nearer to TLC is far more uncomfortable and likely to induce limiting dyspnoea. Thus, it is the combination of both variables that is affected by dynamic hyperinflation; however, it is the IRV that most closely relates to symptom limitation (Guenette et al., 2012).

In our study, we also found that expiratory resistance resulted in a critically low (<0.5 L) IRV (0.19 L on average) and corresponding intolerable dyspnoea. In our control trial participants still achieved a low IRV (Figure 5b) and a similar dyspnoea score to the expiratory resistance trial (Figure 5c). However, the expiratory resistance resulted in a faster decline in IRV and decreased exercise tolerance, as evidenced by our isotime measurements. Our data support the hypothesis that limited inspiratory reserve during exercise, more than expiratory flow limitation or hyperinflation alone, causes intolerable dyspnoea leading to exercise intolerance (Casaburi & Rennard, 2015; Guenette et al., 2012). Furthermore, this dyspnoea may arise due to neuromechanical dissociation between central drive to breathe and lung volume displacement (O'Donnell et al., 2006). Neuromechanical dissociation generates a strong emotional reaction (i.e. fear, distress) in the individual, and at the same time corollary discharge is sent to the motor cortex that may simultaneously reduce the central motor drive to the locomotor muscles (O'Donnell et al., 2007).

### 4.2 What is the effect of imposed expiratory resistance on activation fatigue?

Here, we found greater activation fatigue at isotime with added expiratory resistance (Figure 4b), which may be attributed to the corollary discharge discussed above. This further confirms the important role of central motor drive and/or spinal inhibition in performance fatigue (Amann & Dempsey, 2008). We are unable to determine whether the reduction seen at isotime is due to reduced cortical output, spinal inhibition or motor neuron conduction changes. However, we do know that the relationship between muscle power and muscle activation at a constrained contractile velocity is linear, which allows us to make inferences about the EMG–*P*_iso_ relationship at baseline and intolerance. We found that 90% of the additional performance fatigue with expiratory resistance was attributed to activation fatigue, and 86% was attributed to activation fatigue at isotime. Afferent feedback from fatiguing muscles, increased respiratory work, and receptors in the lung are known to reduce spinal excitability and inhibit motor activation (Undem & Kollarik, 2005; Westerblad & Allen, 2002). While we cannot be sure of the pathway and mechanism of activation fatigue, these are the most likely candidates.

### 4.3 What is the effect of imposed expiratory resistance on skeletal muscle fatigue?

We found no difference in skeletal muscle fatigue (maximal voluntary power at a given EMG activity) at isotime between control and expiratory resistance conditions. In healthy participants, the abnormal lung mechanics and elevated work of breathing did not change the rate of progression of muscle fatigue. As the respiratory muscles become fatigued, blood may be shunted away from the skeletal muscles and towards the diaphragm and accessory muscles of ventilation (Harms et al., 1998). Blood flow may be redistributed via sympathetically mediated vasoconstriction induced by a metaboreflex originating in the respiratory muscles (Harms et al., 1998). Through this mechanism, it is reasonable to expect that imposed expiratory resistance would worsen skeletal muscle fatigue. However, studies of the respiratory blood flow ‘steal’ effect have been inconsistent (Harms et al., 1998; Richardson et al., 1995; Richter et al., 1992). Nevertheless, in our study, consistent with other studies of imposed airway resistance (Kowalchuk et al., 2002), we did not see a direct effect on the skeletal muscle or brain NIRS measures of oxygenation, and no difference in muscle fatigue. We might expect lower cerebral and muscle oxygenation with resistive loading due to demand for respiratory muscle blood flow and the ‘central’ nature of worsened activation fatigue. However, without compelling effects in the cerebral oxygenation in our study, we can only conclude that the cerebrovascular regulatory mechanisms that determine tissue oxygenation are much stronger than any potential manipulation from added expiratory resistive loading.

Changes to the intramuscular biochemical environment contribute to the skeletal muscle fatigue in COPD patients, which may be exacerbated with additional insults to lung mechanics and compromised cardiac output. Interestingly, in a randomized trial of a combination of long‐acting muscarinic antagonist and long‐acting beta‐agonist (LAMA + LABA), neuromuscular fatigue was not affected in COPD patients using a similar measurement approach, despite improved spirometry (Cao et al., 2022).

Little is understood about the interactions between ‘muscle’ and ‘activation’ fatigue or how they contribute to exercise intolerance during whole body exercise; nor is there even consistency in the terminology. Nonetheless, our data support the hypothesis that dynamic hyperinflation and the consequent ventilatory limitations contribute importantly to exercise intolerance through ‘central’ mechanisms, and that dyspnoea is secondary to the dynamic hyperinflation (O'Donnell et al., 2001). However, how these two mechanisms are inter‐dependent is complex enough that only the weakest association exists between our summed measures of dyspnoea and IRV (*r^2^* = 0.11).

### 4.4 Is there a power reserve at the limit of tolerance with imposed expiratory resistance?

We found a power reserve at the limit of tolerance in the ramp incremental (52 (27)%) and the three constant power trials (control = 109 (50)%, expiratory resistance = 107 (55)%, isotime‐control = 136 (44)%; Figure 4d). However, there was more power reserve at isotime‐control compared to expiratory resistance (*P =* 0.09; *n* = 14, CI_diff_ −10, 113 W; Figure 4d). The presence of a power reserve implies the limit of tolerance is not set only by locomotor power limits, and represents the separation between perceptual and physiological limits of cycling power output (Ferguson et al., 2016; Marcora & Staiano, 2010). There was no difference (*F*(2, 33) = 0.18; *P =* 0.84) in ${\dot{V}}_{O_{2}\mathrm{peak}}$ during ramp incremental or constant power trials, so we can be confident that participants attained oxidative system limitations in both formats. It should also be noted that this reserve in locomotor power was only sustained for a brief period (∼3–5 s). The implication of this ‘reserve’ in power is that neither activation fatigue nor muscle fatigue has reached limiting values and therefore they do not, in themselves, limit power output. In other words, at the limit of tolerance, and despite fatigue, the neuromuscular capacity for power output still exceeds the capacity for oxygen transport and/or utilization. We have included more thorough discussions on this issue elsewhere (Baldwin et al., 2024; Cannon et al., 2016; Ferguson et al., 2016; Swisher et al., 2019; Yong et al., 2017).

### 4.5 Conclusions

In conclusion, imposed expiratory resistance initiates a cascade of abnormal lung mechanics, symptoms and reduction in neuromuscular performance, each of which conflates to reduce exercise tolerance. These findings support the hypothesis of a critical IRV accompanying intolerable dyspnoea. The deficit in locomotor power under conditions of dynamic hyperinflation is more likely due to reduced maximal voluntary motor activity than to intrinsic skeletal muscle fatigue.

## AUTHOR CONTRIBUTIONS

Concept and design: Jonathan Cunha, Daniel T. Cannon. Data acquisition: Jonathan Cunha, Daniel T. Cannon. Data analysis: Jonathan Cunha, Antoinette Domingo, Daniel T. Cannon. Figure preparation: Jonathan Cunha, Daniel T. Cannon. Data interpretation: Jonathan Cunha, Antoinette Domingo, Fred W. Kolkhorst, Harry B. Rossiter, Daniel T. Cannon. Manuscript drafting: Jonathan Cunha, Daniel T. Cannon. Critical Revision: Jonathan Cunha, Daniel T. Cannon, Fred W. Kolkhorst, Harry B. Rossiter, Daniel T. Cannon. All authors have read and approved the final version of this manuscript and agree to be accountable for all aspects of the work in ensuring that questions related to the accuracy or integrity of any part of the work are appropriately investigated and resolved. All persons designated as authors qualify for authorship, and all those who qualify for authorship are listed.

## CONFLICT OF INTEREST

H.R. reports consulting fees from the NIH RECOVER‐ENERGIZE working group (1OT2HL156812), and is involved in contracted clinical research with GlaxoSmithKline, Genentech, Intervene Immune, Mezzion, Regeneron, Respira, Roche and United Therapeutics. He is a visiting Professor at the University of Leeds, UK. He reports a pending patent application for the mCPET technique filed by The Lundquist Institute, titled ‘testing system to diagnose neuromuscular deconditioning and pathologic conditions’. The authors have no conflicts of interest.

## Data Availability

All data supporting the results presented in the manuscript are available in a repository https://doi.org/10.6084/m9.figshare.29374037 or https://figshare.com/s/a5d2d2c085783074493d
